# Anti-IL6R role in treatment of COVID-19-related ARDS

**DOI:** 10.1186/s12967-020-02333-9

**Published:** 2020-04-14

**Authors:** Franco Maria Buonaguro, Igor Puzanov, Paolo Antonio Ascierto

**Affiliations:** 1 Melanoma, Cancer Immunotherapy and Development Therapeutics Unit, Istituto Nazionale Tumori IRCCS Fondazione “G. Pascale”, Via Mariano Semmola, 80131 Naples, Italy; 2 Roswell Park Comprehensive Cancer Center, Buffalo, NY USA

**Keywords:** COVID-19, SARS-CoV-2, ARDS, IL-6, Tocilizumab

The COVID-19 pandemic, which has exhausted the momentum in China, is still in the exponential phase in the rest of the world, without even reaching the peak.

What have we learned so far? What did Chinese scientists and doctors teach us?

SARS-CoV-2 infection is not like the seasonal flu. While the range of symptoms for the two viruses is similar, the fraction with severe disease appears to be different. For COVID-19, data to date suggest that 80% of infections are mild or asymptomatic, 15% are severe infection, requiring oxygen and 5% are critical infections characterized by acute respiratory distress syndrome (ARDS), requiring mechanical ventilation. These fractions of severe and critical infection would be higher than what is observed for influenza infection.

There are currently no effective prophylactic or post-exposure therapies. In patients infected with SARS-CoV-2, it has been described that disease severity and outcomes are related to the characteristics of the immune response. Interleukin (IL)-6 and other components of the inflammatory cascade contribute to host defense against infections. However, exaggerated synthesis of IL-6 can lead to an acute severe systemic inflammatory response known as cytokine release syndrome (CRS). In the pathogenesis of SARS-CoV-2 pneumonia, a study found that a CRS involving a considerable release of proinflammatory cytokines occurred, including IL-6, IL-12, and tumor necrosis factor α (TNF-α).

The paper published in this issue by Fu et al., reports preliminary data collected from 21 patients with SARS-CoV-2- induced ARDS treated with tocilizumab [1].

In this single arm study, patients with moderate to severe COVID-19 disease received one or two doses of tocilizumab (400 mg/dose) in addition to standard therapies used including lopinavir and methylprednisolone as reported in the Diagnosis and Treatment Protocol for Novel Coronavirus Pneumonia (6th interim edition) [2]. Most patients experienced clinical improvement including lower oxygen requirement (15/21, 75%), decrease of CRP, increase in lymphocyte levels, decreased fever and improved chest tightness. Two patients were taken off the ventilator within 5 days after the treatment with tocilizumab, another one improved significantly [1].

Based on this data, on March 3rd, 2020, National Health Commission of China included tocilizumab in its 7th edition of COVID-19 therapy recommendations.

Limited experience in Italian centers obtained using tocilizumab for patients with moderate to severe COVID-19 revealed anecdotal evidence of time-correlated clinical improvements in oxygenation, decreased CRP, increased lymphocyte counts 24–48 h post administration, similar to the Chinese experience. Better outcomes were observed in non-intubated patients with elevated baseline level of IL-6 CRP, ferritin and LDH.

Thus, Italian Pharmaceutical Agency (AIFA) approved a Phase II trial in 330 patients with COVID-19 induced ARDS using tocilizumab started on March 19, 2020 (https://www.aifa.gov.it/documents/20142/1127901/TOCIVID-19_Protocol_v1.3_18Marzo2020.pdf/6843930d-9f31-185d-9812-29f02ebebd76) identified in USA as NCT04317092. This is a multicenter, open label, single arm study with primary endpoint of overall mortality 1 month after registration. Secondary endpoints include predictive/prognostic markers of baseline and on treatment level of IL-6 and CRP, lymphocyte count changes, radiological response, and other cytokine changes. Study will include patients with SARS-CoV-2-induced interstitial pneumonia, respiratory insufficiency (O2sat ≤ 93% or PaO2/FiO2 ratio ≤ 300). Patients should be intubated less than 24 h before registration. In parallel, observational cohort with less stringent enrollment criteria will proceed as well.

In the US, an adaptive Phase 2/3, randomized, double-blind, placebo-controlled study assessing efficacy and safety of sarilumab (NCT04315298), another anti-IL-6R antibody started on March 16, 2020, sarilumab binds to both membrane-bound and soluble forms of IL-6R.

The clinical presentation of patients with severe forms of COVID-19 resembles cytokine release syndrome (CRS) observed in some oncology patients treated with CAR-T cell therapies. There, IL-6R inhibition with tocilizumab (anti-IL-6R antibody) proved effective and was FDA approved in 2017. IL-6 and its receptor signaling were shown to play a role in immune response to H1N1 influenza and prevention of lung damage [3–5]. However, administration of tocilizumab has not prevented influenza vaccination immune response in patients with rheumatoid arthritis [6]. its role in patients infected with SARS-CoV-2 has not yet been fully studied and is awaiting completion of clinical trials under way.

Until that time, it seems plausible to speculate that the anti-IL6R mAb plays a protective role if given at the time of overly elevated immune response to the virus, thus preventing “anaphylactic toxicity”. Such extreme cytokine reaction is accompanied by infiltration of inflammatory monocytes/macrophages (IMM) into the lung and elevated production of the pro-inflammatory cytokines (IL-1, IL-6, IL-8, CXCL-10, and MCP-1). These events are associated with severe lung damage, characterized by profound vascular leakage, and death [7]. In this context the anti-IL6R may only be controlling the “cytokine storm” without deleterious effect on virus replication.

## Data Availability

All relevant data are included in the article.

## References

[1] Binqing Fu, Xiaoling Xu, Wei Haiming (2020). Why tocilizumab could be an effective treatment for severe COVID-19?. J Trans Med.

[2] The General Office of National Health Commission Office of State TCM Administration. Diagnosis and Treatment Protocol for Novel Coronavirus Pneumonia (Trial Version 6, Revised). http://www.kankyokansen.org/uploads/uploads/files/jsipc/protocol_V6.pdf. Accessed 18 Feb 2020.

[3] Lauder SN, Jones E, Smart K (2013). Interleukin-6 limits influenza-induced inflammation and protects against fatal lung pathology. Eur J Immunol.

[4] Longhi MP, Wright K, Lauder SN, Nowell MA, Jones GW, Godkin AJ (2008). Interleukin-6 is crucial for recall of influenza-specific memory CD4^+^ T cells. PLoS Pathog.

[5] Dienz O, Rud J, Eaton S (2012). Essential role of IL-6 in protection against H1N1 influenza virus by promoting neutrophil survival in the lung. Mucosal Immunol.

[6] Mori S, Ueki Y, Hirakata N (2012). Impact of tocilizumab therapy on antibody response to influenza vaccine in patients with rheumatoid arthritis. Ann Rheum Dis.

[7] Liu L, Wei Q, Lin Q, Fang J, Wang H, Kwok H, Tang H, Nishiura K, Peng J, Tan Z, Tongjin W, Cheung K-W, Chan K-H, Alvarez X, Qin C, Lackner A, Perlman S, Yuen KY, Chen Z (2019). Anti–spike IgG causes severe acute lung injury by skewing macrophage responses during acute SARS-CoV infection. JCI Insight..

